# Fluoroquinolone resistance does not facilitate phage Φ13 integration or excision in *Staphylococcus aureus*

**DOI:** 10.1099/acmi.0.000583.v4

**Published:** 2023-06-09

**Authors:** Helena Leinweber, Raphael N. Sieber, Martin S. Bojer, Jesper Larsen, Hanne Ingmer

**Affiliations:** ^1^​ Department of Veterinary and Animal Sciences, University of Copenhagen, Stigbøjlen 4, 1870 Copenhagen, Denmark; ^2^​ Department of Bacteria, Parasites and Fungi, Statens Serum Institut, Artillerivej 5, 2300 Copenhagen, Denmark

**Keywords:** Sa3int phage, *S. aureus*, supercoiling, fluoroquinolone resistance, integration

## Abstract

Prophages of the ΦSa3int family are commonly found in human-associated strains of *Staphylococcus aureus* where they encode factors for evading the human innate immune system. In contrast, they are usually absent in livestock-associated methicillin-resistant *S. aureus* (LA-MRSA) strains where the phage attachment site is mutated compared to the human strains. However, ΦSa3int phages have been found in a subset of LA-MRSA strains belonging to clonal complex 398 (CC398), including a lineage that is widespread in pig farms in Northern Jutland, Denmark. This lineage contains amino acid changes in the DNA topoisomerase IV and the DNA gyrase encoded by *grlA* and *gyrA*, respectively, which have been associated with fluoroquinolone (FQ) resistance. As both of these enzymes are involved in DNA supercoiling, we speculated that the mutations might impact recombination between the ΦSa3int phage and the bacterial chromosome. To examine this, we introduced the FQ resistance mutations into *S. aureus* 8325-4*attB_LA_* that carry the mutated CC398-like bacterial attachment site for ΦSa3int phages. When monitoring phage integration and release of Φ13, a well-described representative of the ΦSa3int phage family, we did not observe any significant differences between the FQ-resistant mutant and the wild-type strain. Thus our results suggest that mutations in *grlA* and *gyrA* do not contribute to the presence of the ΦSa3int phages in LA-MRSA CC398.

## Data Summary

Sequence data have been placed and are available at the European Nucleotide Archive (https://www.ebi.ac.uk/ena/browser/home) under bioproject PRJEB46987. From these single nucleotide polymorphism (SNP) were called using the NASP pipeline. Recombination was removed from the SNP alignment using Gubbins [1] and the remaining SNPs in the core genome were used to construct a maximum-likelihood tree using IQ-TREE version 2.0.3. Statistical analysis was done in R version 4.0.3(10.10.2020) using package epitools version 0.5–10.1.

## Introduction

*Staphylococcus aureus* is an opportunistic bacterial pathogen that colonizes about one-third of the human population in addition to a wide range of animals [2]. Staphylococcal strains are divided into lineages with some being livestock-associated (LA) while others are hospital or community associated. Despite the preference of LA-strains for livestock, a significant fraction of human infections are caused by LA-strains that are resistant to methicillin (LA-MRSAs) and close contact with livestock is a risk factor [3, 4].

Host specificity of *S. aureus* is tightly linked to its content of mobile genetic elements [5]. Most human strains carry prophages of the ΦSa3int family that are integrated in the bacterial attachment site (*attB*) located in the *hlb* gene [6]. ΦSa3int phages carry an immune evasion cluster (IEC) that encodes one or more immune evasion factors and their presence promotes human colonization and human-to-human transmission of *S. aureus* [7–9]. In contrast, LA-MRSA strains belonging to the clonal complex CC398 commonly lack ΦSa3int phages. This may be related to their variant phage attachment site (designated *attB_LA_*) [7, 10, 11] where the sequence 5′-TGTATCCGAATTGG-3′ differs from non-livestock, human strains at the underlined nucleotides [10, 11]. These nucleotide changes decrease the integration frequency of ΦSa3int phages and lead to integration at other locations in the bacterial genome than the *hlb* gene [10–14]. Despite the reduced integration frequency there is an overrepresentation of strains with ΦSa3int phages in human isolates of CC398 compared to livestock isolates [8]. Also the presence of the prophage is associated with ‘spillover’ events where humans are infected with LA-MRSA CC398 strains without having had livestock contact, indicating an increased risk of human-to-human transmission [15, 16]. This is especially problematic in areas with intensive pig farming where those strains have become a serious human disease burden [17, 18].

**Fig. 1.**
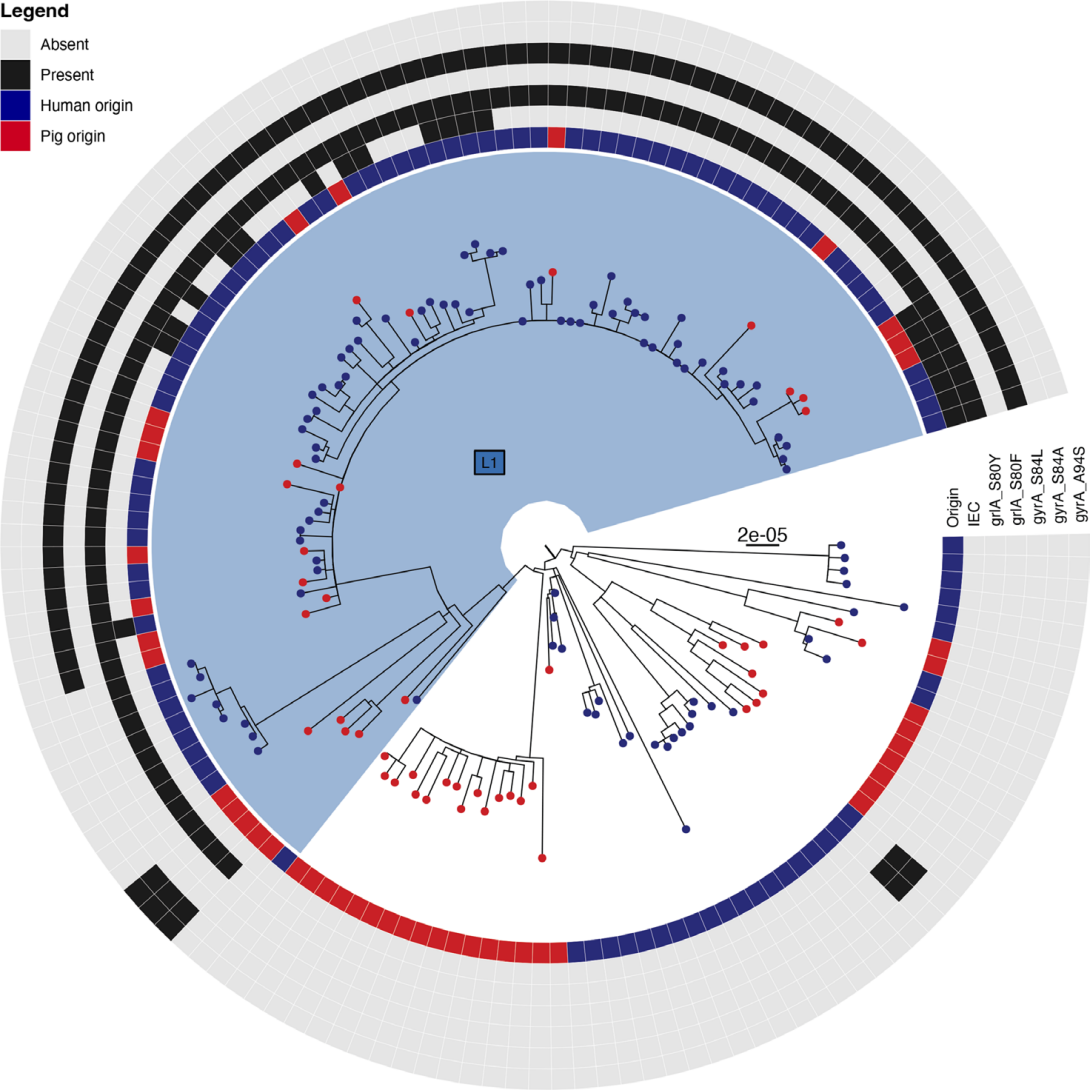
Phylogenetic tree of 141 LA-MRSA CC398 strains isolated from either humans (96, dark blue) or pigs (45, red) form Northern Jutland, Denmark. Presence (black) and absence (light grey) of the IEC and Sa3int phage as well as grlA and gyrA mutations conferring fluoroquinolone resistance is indicated. The tree was rooted according to [6] and the scale bar represents the number of nucleotide substitutions per variable site.

We recently identified and characterized 20 LA-MRSA CC398 strains from Northern Jutland, Denmark with 17 isolates obtained from humans and three from pigs, that all harboured IEC-carrying ΦSa3int prophages [13]. The prophages could be divided into six variants on the basis of their phylogenetic relationship, IEC type, and chromosomal integration site and each variant was unique to a single household. Interestingly, all isolates exhibited decreased susceptibility to fluoroquinolones (FQ) and belonged to the same lineage, L1 (Fig. 1). As these findings indicated that there had been several integration events by ΦSa3int phages, we wondered if there may be a link between FQ resistance and ΦSa3int phage integration or stability.

FQs are a class of synthetic broad-spectrum antibiotics, and newer generations of this class are effective against a broad spectrum of Gram-negative and -positive bacteria, including *S. aureus* [19]. In humans they are frequently prescribed [20], whereas in livestock, FQs are tightly regulated and only prescribed after laboratory susceptibility testing [21]. FQs primarily target the A-subunits of the bacterial DNA topoisomerase IV and the DNA gyrase encoded by *grlA* and *gyrA*, respectively [22]. Both enzymes shape DNA topology through cleavage, entwinement and re-ligation of DNA double strands. Binding of FQ at the enzyme-DNA intersection leads to stalling of DNA re-ligation and synthesis, which ultimately results in cell death [23].

FQ resistance is mediated by mutations clustered in the 5′-end of *gyrA* (between residues 68–107) and *grlA* (between residues 64–103) [24, 25] leading to reduced binding of FQ at the catalytic sites. When *S. aureus* is exposed to FQ*,* mutations occur stepwise with *grlA* being modified first leading to a 64-fold increase in the minimal inhibitory concentration (MIC) to ciprofloxacin from 0.5 µg ml^−1^ to 32 µg ml^−1^. In a subsequent step, mutations in *gyrA* confer high level resistance with an increase in MIC to 128 µg ml^−1^ [25].

The structural changes of DNA-modifying enzymes associated with FQ resistance may also affect DNA supercoiling. This has been seen in *Campylobacter jejuni* [26], *Pseudomonas aeruginosa* [27] and *Escherichia coli* [28]. Importantly, supercoiling can influence phage integration as observed for the *E. coli* phage λ, where binding of the phage integrase to the phage attachment site (*attP*) was affected by supercoiling [29]. Further, the DNA damage imposed by FQs can lead to induction of prophages through activation of the SOS response [30, 31]. This entails activation of RecA and cleavage of the SOS repressor LexA, as well as a number of phage repressors that ensure lysogeny in temperate phages. Prophage induction by FQs has been observed for several phages in for example *Salmonella enterica* serovar Typhimurium [30], *Streptococcus pneumoniae* [32] and *S. aureus* [33, 34].

To examine whether there may be a link between FQ resistance in LA-MRSA CC398 strains and lysogeny of ΦSa3int phages, we introduced the *gyrA/grlA* mutations in *S. aureus* strain 8325-4*attB_LA_*, that is a derivative of 8325–4 carrying the CC398-like bacterial attachment site (*attB_LA_*) [11]. Subsequently we compared lysogenization frequencies and prophage induction of the ΦSa3int phage Φ13. Our results show that the FQ resistance mutations did not affect ΦSa3int phage integration or prophage stability.

## Methods

### Strains and growth conditions

An overview of the strains used can be found in Table 1. *S *. *aureus* was grown and plated on tryptic soy broth or agar medium (TSB/TSA), containing 6 µg ml^−1^ ciprofloxacin (Cip) and/or 30 µg ml^−1^ kanamycin (Kan) if appropriate. *E. coli* was grown and plated on Luria-Bertani medium (LB) with 100 µg ml^−1^ ampicillin.

**Table 1.** Strains and plasmids used in this study

Strain name	Species	Description	Reference
8325-4*attB_LA_*	* S. aureus *	8325–4 mutated at ɸ13 attB site in *hlb* (designated *attB_LA_), hlb*+	[11]
8325-4ɸ13kan^R^	* S. aureus *	8325–4 lysogenized with ɸ13kan^R^, *hlb*-, kanamycin resistant	[11]
8325-4*attB_LA_* FQ^R^	* S. aureus *	8325-4attB_LA_containing mutation Ser80 → Tyr80 in *grlA* and Ser84 → Leu84 in *gyrA*, conferring resistance to ciprofloxacin	This study
CC398	* S. aureus *	Strain collection of 141 LA-MRSA CC398 strains from an outbreak in Denmark	[13]
IM08B	* E. coli *	K12 DC10B expressing *S. aureus* CC8 type I methylation	[36, 46]
pBASE6		*E. coli – S. aureus* temperature-sensitive suicide shuttle vector, Amp^R^, Cm^R^	[35]

### Strain constructions

The *gyrA* and *grlA* mutations conferring FQ resistance found in LA-MRSA CC398 were introduced in *S. aureus* 8325-4*attB_LA_* using pBASE6 [35] and via *E. coli* IM08B [36]. *S. aureus* 8325-4*attB_LA_* carries a 2 bp mutation in the bacterial attachment site in the *hlb*-gene, found in LA-* S. aureus* CC398 [11]. The genome sequence of *S. aureus* NCTC8325 (GenBank Accession no. NC_007795.1) was used as a template for construction of ca. 2 kb fragments containing the respective point mutations (*grlA* Ser80 → Tyr and *gyrA* Ser84 → Leu) with restriction sites at each end (purchased from Twist Bioscience) and amplified with primers and conditions stated in Table 2. The fragments were cloned into pBASE6 using BglII (AGATCT) and KpnI (GGTACC) and the resulting plasmids pBASE6:grlA and pBASE6:gyrA were purified using GeneJET Plasmid Mini Prep kit (Thermo Scientific). In a first step, pBASE6:grlA was transformed into *S. aureus* 8325-4*attB_LA_* by electroporation (2.1 kV cm^−1^, 100 Ω, 25 µF), followed by plating on TSA with 10 µg ml^−1^ chloramphenicol (Cm). After incubation, one colony was selected, grown in TSB with 10 µg ml^−1^ Cm and 10-fold dilutions were plated on TSA with 2 µg ml^−1^ Cip to obtain 8325-4*attB_LA_*:grlA. One colony was selected, cultured in TSB with 2 µg ml^−1^ Cip and made competent [37]. In a second step, 8325-4*attB_LA_*:grlA served as recipient for pBASE6:gyrA and plating was done at 8 µg ml^−1^ Cip, to obtain the final strain containing both mutations in *grlA* and *gyrA*, designated 8325-4*attB_LA_*:FQ^R^. As *gyrA/grlA* are essential genes we decided to try a double cross in/out event in a single step and simply select by Cip. Spontaneous loss of vector pBASE6 was confirmed by lack of growth on TSA with 10 µg ml^−1^ chloramphenicol. DNA was isolated using DNeasy Blood and Tissue kit (Qiagen) with pretreatment for Gram-positive bacteria and presence of both mutations was confirmed using 251 bp paired-end sequencing on an Illumina MiSeq machine (Illumina, San Diego, California, United States). Sequence data is available at the European Nucleotide Archive (https://www.ebi.ac.uk/ena/browser/home) under bioproject PRJEB46987.

**Table 2.** Primers and PCR conditions for amplification of cloning inserts using Phusion Hot Start II High-Fidelity DNA Polymerase 2 x Mastermix (Thermo Scientific)

Primer name	Sequence 5´- 3´	Annealing temp. °C	Elongation time (s)	Reference
*Grl-fwd*	GATACAAGATCTGATATGCATACG	62	30	This study
*Grl_rev*	ATATGGTACCGCGATATTACCATTACGTGG			
*Gyr_fwd*	GATACAAGATCTGTGGGCACG	66	30	This study
*Gyr_rev*	ATATGGTACCCTGGTCGTGACTCTAGAACG			

### Phylogenetic tree construction

For phylogenetic reconstruction, raw sequence reads from the previous study [16] were mapped against LA-MRSA CC398 strain S0385 (GenBank accession no. NC_017333), and single nucleotide polymorphism (SNP) were called using the NASP pipeline [38]. Recombination was removed from the SNP alignment using Gubbins [1] and the remaining SNPs in the core genome were used to construct a maximum-likelihood tree using IQ-TREE version 2.0.3 [1]. Previously published metadata on genomic elements was used [16], and Mykrobe Predictor version 0.5.6 [37] was used to identify FQ-resistance causing point mutations. The phylogeny was visualized by using the R-package ggtree version 2.4.1 [39].

### Susceptibility testing

All 96 previously identified human LA-MRSA CC398 isolates from North Jutland, Denmark [13] were tested for resistance to norfloxacin by use of the disc diffusion method, in accordance with the European Committee on Antimicrobial Susceptibility Testing guidelines [40]. Minimal inhibitory concentration (MIC) for Cip was determined with microbroth dilution method [41]. Cip (Sigma Aldrich) was diluted to span a range between 256 and 0.5 µg ml^−1^ in the columns of a 96-well plate. Bacteria were added to the wells at a concentration of 1×10^5^ c.f.u. ml^−1^ and MIC was determined as the lowest concentration without growth after overnight incubation at 37 °C.

### Growth assessment

Doubling times were assessed by OD_600nm_ measurement in Bioscreen C MBR (Oy Growth Curves Ab Ltd). For this, 300 µl of the respective culture (starting OD_600nm_=0.05) was added to each well followed by overnight incubation with shaking at 37 °C. OD_600nm_ was measured every 20 min. Doubling time was calculated by using the programme Growthrates 3.0 [42].

### Lysogenization assay

Lysogenization with Φ13kan^R^ was performed as described previously [11] and frequencies were calculated for 8325-4*attB_LA_* by dividing the number of colonies on TSA +Kan by the number of colonies on TSA and for 8325-4*attB_LA_*:FQ^R^ by dividing the number of colonies on TSA +Kan+ Cip by the number of colonies on TSA +Cip.

### Phage release

To determine phage release we selected lysogens with Φ13kan^R^ in *attB_LA_* in the *hlb* gene. The cultures were freshly grown until OD_600nm_=1 and either with or without the addition of 2 µg ml^−1^ Mitomycin C incubated for 2 h at 37 °C and 180 r.p.m. [11, 12]. The cultures were centrifuged (5 min, 9000 r.p.m.), the supernatant sterile filtered using 0.2 µm membrane filters, 10-fold diluted in SM-buffer (100 mM NaCl, 50 mM Tris [pH=7.8], 1 mM MgSO_4_, 4 mM CaCl_2_) and spotted on a lawn of *S. aureus* 8325–4. The lawn was prepared by adding 100 µl fresh culture (OD_600nm_=1) to 3 ml top agar (mix of TSA (one part) and TSB (four parts) and 10 µM CaCl_2_).

### Statistical analysis

Statistical analysis was done in R version 4.0.3 (10.10.2020) [43] using package epitools version 0.5–10.1 [44].

## Results and discussion

While analysing a subset of LA-MRSA CC398 strains isolated from pigs (*n*=45) and humans (*n*=96) in Northern Jutland, Denmark [13], we noticed that all isolates harbouring a ΦSa3int prophage were also resistant to FQ and had mutations in *gyrA*/*grlA* (Fig. 1 and Table 3). There was a significantly greater carriage of a ΦSa3int phage in strains containing at least one of these mutations than in the remaining strains (0/26 [FQ-sensitive] versus 17/70 [FQ-resistant], fisher’s exact *P*=0.0049). To investigate if the *gyrA*/*grlA* mutations influence interactions between ΦSa3int phages and LA-MRSA CC398 strains, we introduced the FQ resistance mutations into a phage-cured laboratory strain carrying *attB_LA_* resulting in *S. aureus* 8325-4*attB_LA_*:FQ^R^ that had a MIC of 8 µg ml^−1^ Cip compared to 0.5 µg ml^−1^ for 8325-4*attB_LA_*.

**Table 3.** Mutations of *gyrA* and *grlA* found in *S. aureus* CC398 isolates compared to *S. aureus* NTCT8325 (GenBank Accession no. NC_007795.1)

*grlA*		*gyrA*	
Mutation	Amino acid change	Mutation	Amino acid change
C → A	Ser80 → Tyr	C → T	Ser84 → Leu
C → T	Ser80 → Phe	C → G	Ser84 → Ala
G → A	Val590 → Ile	G → T	Ala94 → Ser
G → A	Val656 → Ile	A → C	Ala772 → Val
		A → C	Glu815 → Asp
		A → T	Glu887 → Asp
		T → G	*888 → Glu

**Fig. 2.**
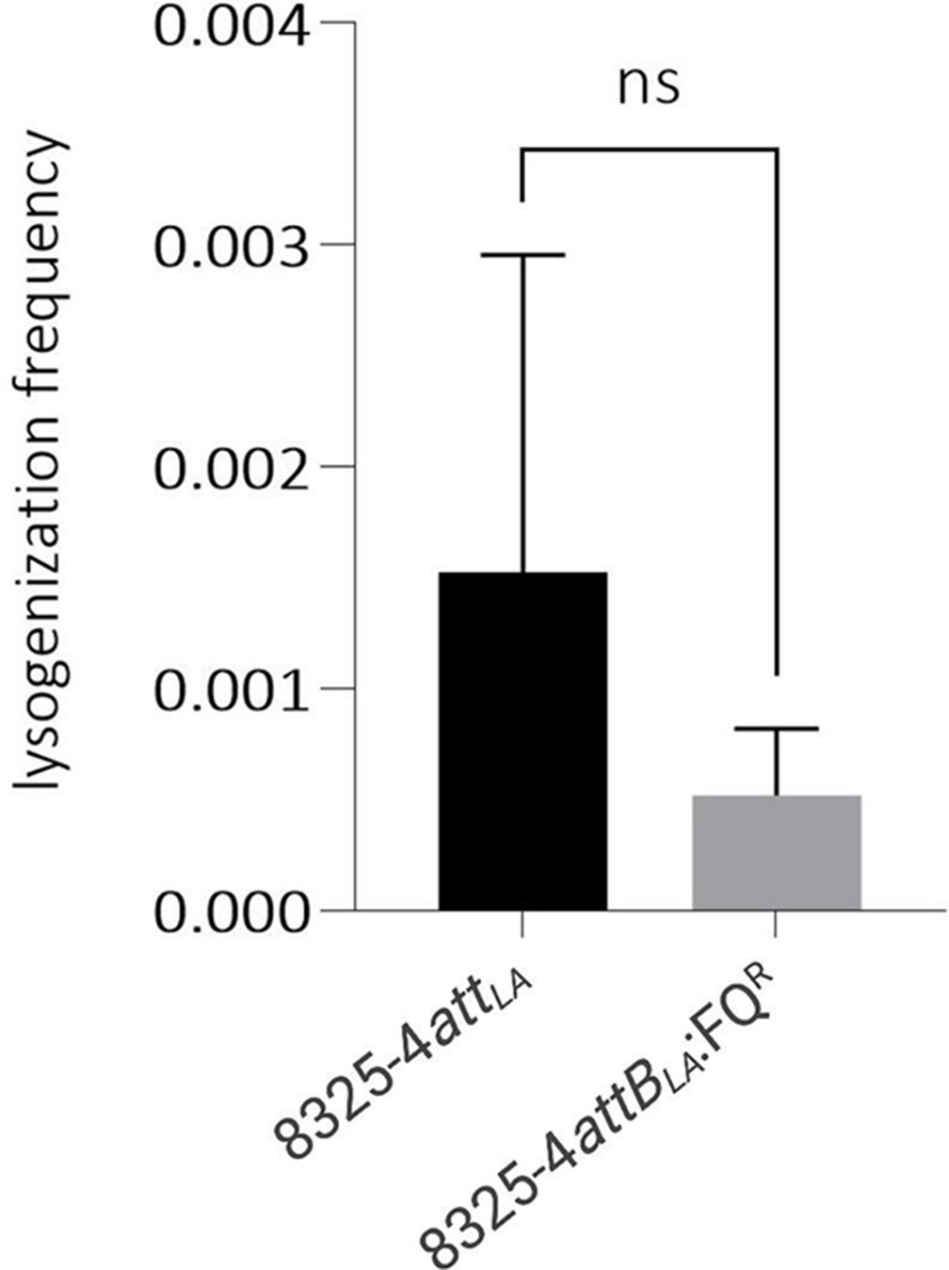
Lysogenization frequencies of ɸ13kan^R^ in *S. aureus* 8325-4attB_LA_ and 8325-4attB_LA_:FQ^R^. Error bars represent standard deviation from six replicates. Significance was determined using Mann-Whitney’s t-test, *P*=0.2403= not significant.

**Fig. 3.**
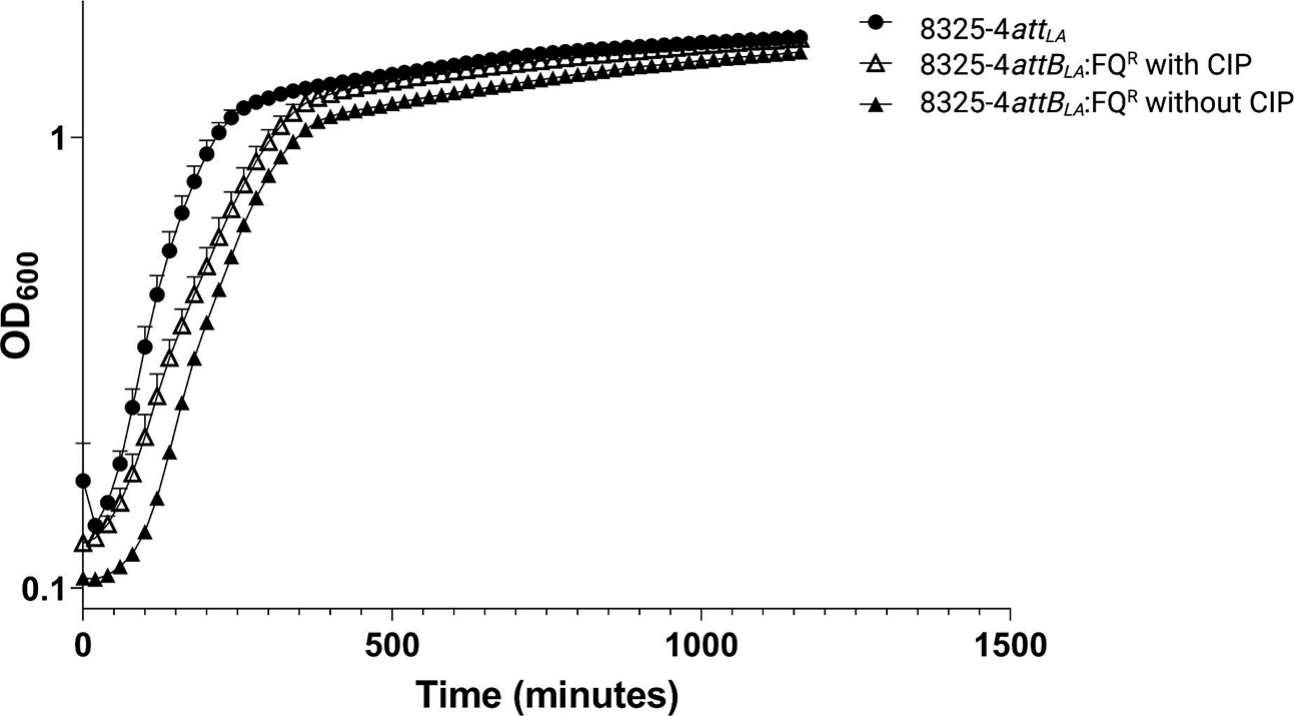
Growth curves of *S. aureus* 8325-4*attB*_*LA*_ and its mutant 8325-4*attB*_*LA*_:FQ^R^. Experiment has been carried out in Bioscreen with growth in TSB or TSB with 6 µg ml^−1^ Cip at at 37 °C, shaking for 1160 min. Error bars represent standard deviation of three biological replicates, with three technical replicates each.

To examine the impact of the *grlA*/*gyrA* mutations on ΦSa3int phage integration, we infected 8325-4*attB_LA_* and 8325-4*attB_LA_*:FQ^R^ with a derivative of the ΦSa3int phage Φ13 that harbours a kanamycin resistance gene (Φ13kan^R^ [11]) at a multiplicity of infection of one and monitored the number of kanamycin-resistant colonies arising (Fig. 2). We found no significant difference in the ability of Φ13kan^R^ to integrate in the two strains and thus we conclude that the *gyrA*/*grlA* mutations do not affect the frequency with which ΦSa3int phages integrate. The slightly lower lysogenization frequency observed in *S. aureus* 8325-4*attB_LA_*:FQ^R^ could be due to an increased doubling time of 41 min compared to 30 min for 8325-4*attB_LA_* (Fig. 3). However, the colony forming units on kanamycin-free medium were similar for wild-type and mutant cells under the conditions used for lysogenization (Table 4).

**Table 4.** Lysogenization of 8325-4attB_LA_ and 8325-4attB_LA_:FQ^R^ with Φ13kan^R^

	c.f.u.		no. of lysogens	
	8325-4*attB_LA_* ^∗^	8325-4*attB_LA_*:FQ^R^†	8325-4*attB_LA_* ‡	8325-4*attB_LA_*:FQ^R^§
**replicate 1**	2.10E+08	2.00E+08	5.90E+04	1.50E+05
**replicate 2**	2.50E+08	1.10E+08	5.60E+05	9.60E+04
**replicate 3**	1.90E+08	2.30E+08	8.50E+03	1.60E+03
**replicate 4**	1.80E+08	9.90E+07	2.00E+05	5.20E+04
**replicate 5**	2,.10E+08	2.00E+08	8.20E+05	1.00E+05
**replicate 6**	1.90E+08	1.10E+08	3.10E+05	5.10E+04

*c.f.u. per ml on TSA.

†c.f.u. per ml on TSA +6 µg ml^−1^ Cip.

‡c.f.u. per ml on TSA +30 µg ml^−1^ Kan.

§c.f.u. per ml on TSA +30 µg ml^−1^ Kan+6 µg ml^−1^ Cip.

**Fig. 4.**
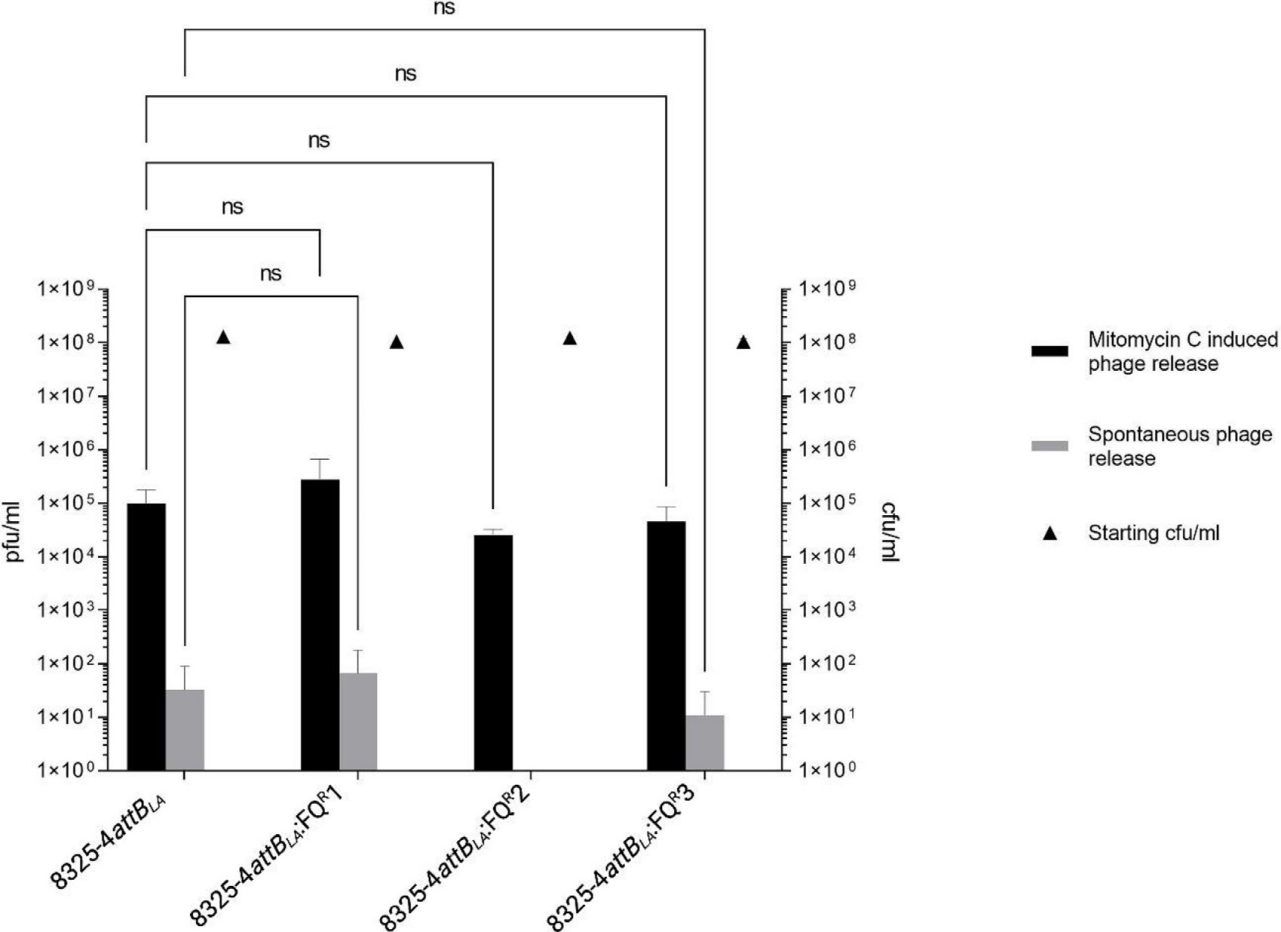
Spontaneous (grey bars) and mitomycin C induced phage release (black bars). Cultures were grown to OD_600nm_=1 at which the starting cfu ml^−1^ was determined (black triangles). From then on, cultures were incubated for another 2 h either without or with the addition of 2 µg ml^−1^ mitomycin C.

We further investigated whether the *grlA*/*gyrA* mutations influence spontaneous or mitomycin-induced prophage release from a lysogen. To this end, we monitored lysogens of 8325-4*attB_LA_* and 8325-4*attB_LA_* FQ^R^ with Φ13kan^R^ integrated as a prophage in *attB_LA_*. As shown in Fig. 4, no significant difference was found between the FQ-resistant or -sensitive isolates from three separate lysogens with three replicates each, both in regards to spontaneous or mitomycin C-induced phage release. Lysogen 8325-4*attB_LA_* FQ^R^2 did not show spontaneous phage release, which may be due to the generally low number of released phages and the insensitivity of the assay. These data suggest that FQ resistant strains are not a favoured reservoir of ΦSa3int phages which otherwise could have explained why FQ resistant strains harbouring ΦSa3int phages were isolated from humans. However, it should be noted that Φ13 only shows up to 66 % sequence identity with the ΦSa3int phages found in the 17 CC398 strains. So even though the phages belong to the phage family with a Sa3 integrase [45] we cannot rule out that there may be phage specific factors encoded by the ΦSa3int phages of the CC398 strains which influence the phage integration/excision processes and are missing in Φ13. Detailed studies will be needed to examine this further.

In conclusion, with the ΦSa3int phage Φ13 and the *S. aureus* 8325-4*attB_LA_* model strain we did not observe an apparent link between FQ resistance and integration or stability of the phage. Future studies should be directed at unravelling the mechanisms leading to the frequent acquisition of IEC-carrying ΦSa3int phages by different LA-MRSA CC398 lineages to prevent further spread into the general population.
